# *Ashwagandha*‐induced acute liver injury: A case report

**DOI:** 10.1002/ccr3.7078

**Published:** 2023-03-14

**Authors:** Marcell Tóth, Andrea E. Benedek, Thomas Longerich, Helmut‐Karl Seitz

**Affiliations:** ^1^ Institute of Pathology University Hospital Heidelberg Heidelberg Germany; ^2^ Department of Medicine Salem Medical Center Heidelberg Germany

**Keywords:** ashwagandha, drug‐induced liver injury, herbal and dietary supplements

## Abstract

Drug‐induced liver injury became one of the most important liver disorders and diagnostic challenge for clinicians and pathologists. Here, we report a rare case of *ashwagandha*‐induced acute liver injury. The patient developed jaundice 2 weeks after starting *ashwagandha* intake and recovered within 5 months after withdrawal without any specific treatment.

## Graphical abstract

Liver biopsy revealing prominent hepatocellular and canalicular cholestasis as well as spotty hepatocellular necrosis with ceroid‐laden macrophages caused by *ashwagandha* intake.

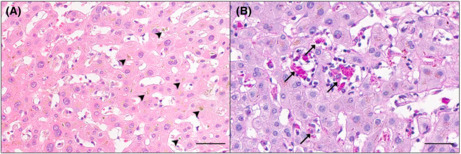

## 1 INTRODUCTION

A wide variety of drugs may cause a broad spectrum of liver disease (drug‐induced liver injury; DILI), varying from asymptomatic elevation of liver enzymes to acute liver failure. DILI associated with the use of conventional or herbal dietary supplements (HDS) became one of the most common causes of acute liver failure worldwide.^1^ *Withania somnifera* or *ashwagandha* (also known as Indian ginseng or winter cherry) is a commonly used herb of the traditional Indian (Ayurvedic) medicine. In addition, it is also marketed as dietary supplement in the Western countries.^2^ Despite its global use, only a few cases of associated liver injuries have been reported.^3^, ^4^, ^5^ Here, we report a rare case of *ashwagandha*‐induced acute liver injury.

## 2 CASE REPORT

A 65‐year‐old woman (164 cm; 69 kg) was referred to the Department of Gastroenterology, Salem Medical Centre, Heidelberg, due to suspected toxic hepatitis. On admission, the patient had jaundice of the skin and sclera without any abdominal pain. She reported a weight loss (3 kg in 3 weeks) as well as diarrhea (about 4 times/day) with pale stool and dark urine during the last 3 weeks.

The patient was in good general condition without any known history of liver disease or jaundice before. The patient's medical history included arterial hypertension (long‐term treatment with phytotherapeutic product named *Rauwolfia serpentina*), psoriasis, and a status post‐tonsillectomy, without any records of complications.

She reported no consumption of alcohol and no history of smoking. The patient traveled to the Mediterranean Sea for holidays 3 months before her presentation in the hospital. The patient experienced no physical symptoms, such as fever or gastrointestinal abnormalities during or following the holiday. She reported only a herpes labialis, which was treated by acyclovir orally.

She began to take an herbal extract, named *ashwagandha*, because of “troubled thoughts” 4 weeks before admission. She stopped intake of the drug after the onset of the above‐mentioned symptoms about 2 weeks before admission. At that time, her blood tests were abnormal. The dosing of *ashwagandha* was not recorded.

The laboratory test at admission showed severely elevated serum cholestasis parameters (total bilirubin 17.3 mg/dL and alkaline phosphatase (AP) 298 U/L; Table 1). Ultrasound examination revealed regular findings, in particular no hepatomegaly and no intra‐ or extrahepatic cholestasis. The liver stiffness was increased to the upper limit (11.5 kPa), the controlled attenuated parameter as determined by transient elastography (Fibroscan, Echosens, Paris) was within the normal range (210 dB/m).

**TABLE 1:** Summary of the laboratory values.

	Ref.	‐2 W	‐1 W	Adm	D 1	D 2	D 3	D 4	D 5	D 6
GOT (U/L)	<35	38	45	41	39	37	40	51	44	43
GPT (U/L)	<35	82	67	54	48	45	46	53	49	50
GGT (U/L)	<40	20	21	18	16	14	17	16	14	14
AP (U/L)	35–105	247	282	298	299	278	284	290	267	271
Bili (mg/dL)	<1.2	12.40	19.15	17.3	16.5	16.4	16.9	‐	15.3	15.6

Abbreviations: ‐2 W and ‐1 W, 2 weeks and 1 week before admission; Adm, day of admission; AP, alkaline phosphatase; Bili, total bilirubin; .D 1 to D 6, first to sixth day after admission; GGT, γ‐glutamyltransferase; GOT, glutamic oxaloacetic transaminase; GPT, glutamate‐pyruvate transaminase; Ref, reference value.

**FIGURE 1:**
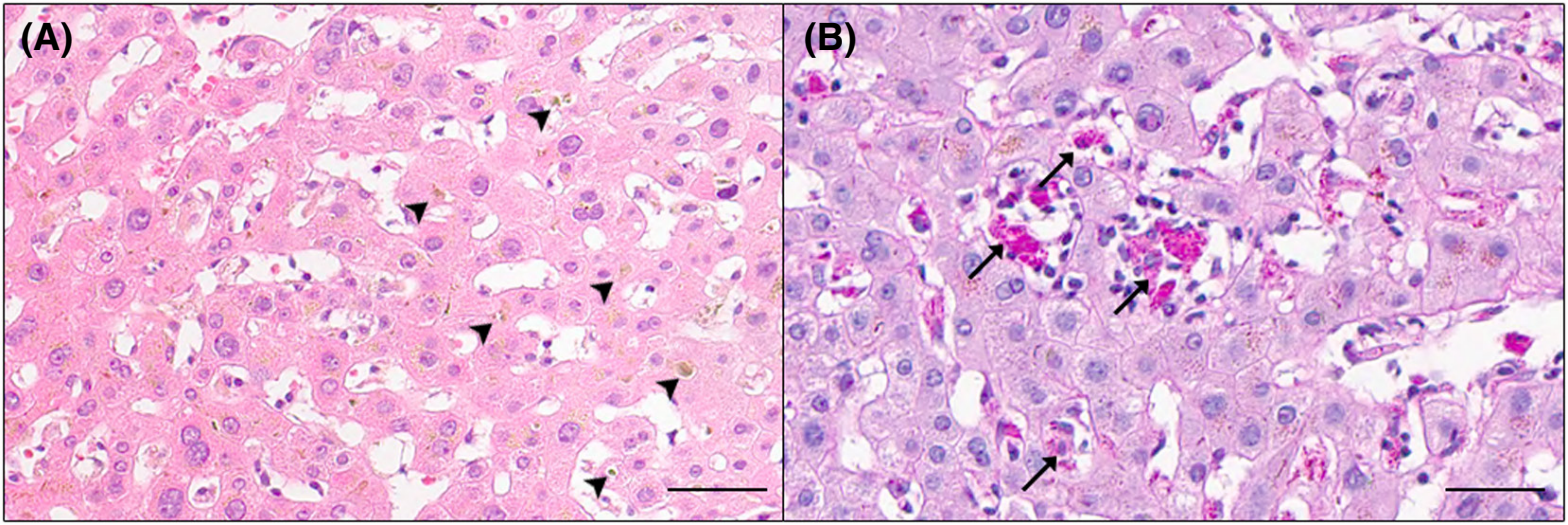
Liver biopsy 4 days after admission (A) Representative hematoxylin and eosin stain of the liver biopsy shows a prominent hepatocellular and canalicular cholestasis. Cholestatic changes are marked with arrowheads. (B) There is a spotty hepatocellular necrosis with ceroid‐laden macrophages in the perivenular parenchyma in the PAS diastase stain. Ceroid‐laden macrophages are demonstrated by arrows. Bars: 50 μm.

Infectious and autoimmune causes of abnormal liver tests were excluded. Serology for viral hepatitis (HAV, HBV, HCV, and HEV) and cytomegalovirus was negative. No auto‐antibodies were detected. 4 days after admission, a liver biopsy was performed and revealed a regular architecture with inconspicuous portal tracts containing scattered ceroid‐laden macrophages. The perivenular parenchyma showed spotty hepatocellular necrosis, accompanied with multiple ceroid‐laden macrophages, hepatocellular, and canalicular cholestasis and features of hepatocellular regeneration, favoring a drug‐induced liver injury (Figure 1).

The only drug or toxin exposure in the patient's history prior to the admission that could explain the observed laboratory findings and the histological picture was *ashwagandha*.

Following withdrawal of the drug, the serum transaminase activities and serum bilirubin gradually dropped without any further specific therapy (Table 1). Only a symptomatic therapy with cholestyramine was administered on the second day due to pruritus.

The patient could be discharged on the seventh day after admission in good general condition. During follow‐up, the elevated liver parameters gradually normalized—expect for a slight elevation of total bilirubin (1.4 mg/dL)—2 months after discharge from hospital. 3 months later, all liver tests were normal and the liver stiffness was back within reference range (4.3 kPA).

## 3 DISCUSSION

*Ashwagandha* is a widely used herbal extract available over‐the‐counter for a variety of rheumatological conditions (e.g., arthritis), to increase energy levels, improve overall health, and prevent diseases.^2^ Here, we report a rare case of *ashwagandha*‐induced liver injury. Similar to the previously reported cases, our patient also had a marked hyperbilirubinemia and the liver biopsy displayed a prominent canalicular cholestasis.^3^, ^4^, ^5^ Among the liver tests, the cholestatic parameters were leading (total bilirubin 17.3 mg/dL, AP 298 U/L; Table 1), while transaminases showed slightly increased levels (GOT 41 U/L, GPT 54 U/L). In our case, the patient developed jaundice 2 weeks after starting *ashwagandha* intake and recovered within 5 months after withdrawal without any specific treatment.

The patient had a herpes simplex virus infection, which was treated by acyclovir, 3 months prior her admission to the hospital. Hepatic involvement is a rare complication of herpes simplex infection, varying from asymptomatic to acute liver failure. However, herpes hepatitis is characterized by confluent hemorrhagic necrosis, destruction of the reticulin network, and presence of nuclear inclusion bodies. Furthermore, liver involvement in herpes simplex infection is usually anicteric.^6^, ^7^ Although acyclovir is a widely used antiviral drug, there are only a few cases reported, where oral acyclovir therapy caused apparent liver injury.^8^, ^9^

The patient also reported a long‐term usage of the phytotherapeutic product *Rauwolfia serpentina*. Although there is no available literature about the hepatotoxicity of *Rauwolfia serpentina*, the prolonged usage of the product makes it in our case unlikely as the cause of the liver damage. No further possible hepatotoxic agent was detected and the liver biopsy excluded any underlying co‐existent liver pathology.

## 4 CONCLUSION

As a plethora of HDS could cause DILI, the careful acquisition of the medication history, including nonprescription medication or dietary supplements, is an important part of establishing the diagnosis in cases of apparent liver injury without preconditions, such as underlying liver disease of other origins.

## AUTHOR CONTRIBUTIONS

**Marcell Tóth:** Conceptualization; investigation; visualization; writing – original draft. **Andrea Eszter Benedek:** Conceptualization; investigation; writing – original draft. **Thomas Longerich:** Conceptualization; investigation; writing – review and editing. **Helmut‐Karl Seitz:** Conceptualization; investigation; writing – review and editing.

## FUNDING INFORMATION

For the publication fee we acknowledge financial support by Deutsche Forschungsgemeinschaft within the funding program “Open Access Publishing Fund” as well as by Heidelberg University.

## CONFLICT OF INTEREST STATEMENT

None.

## CONSENT

The patient provided written consent for her clinical data to be used for scientific presentations or publications.

## Data Availability

No data available.
